# Can We Predict Preterm Delivery Based on the Previous Pregnancy?

**DOI:** 10.3390/jcm10071517

**Published:** 2021-04-05

**Authors:** Tamar Wainstock, Ruslan Sergienko, Eyal Sheiner

**Affiliations:** 1 Department of Public Health, Faculty of Health Sciences, Ben-Gurion University of the Negev, Beer-Sheva 8489325, Israel; sergienk@bgu.ac.il; 2 Department of Obstetrics and Gynecology, Soroka University Medical Center, Ben-Gurion University of the Negev, Beer-Sheva 8489325, Israel; sheiner@bgu.ac.il

**Keywords:** perinatal mortality, preeclampsia, pregnancy complications, preterm birth, preterm delivery, small for gestational age

## Abstract

(1) Background: Preterm deliveries (PTD, <37 gestational weeks) which occur in 5–18% of deliveries across the world, are associated with immediate and long-term offspring morbidity, as well as high costs to health systems. Our aim was to identify risk factors during the first pregnancy ending at term for PTD in the subsequent pregnancy. (2) Methods: A retrospective population- based nested case−control study was conducted, including all women with two first singleton consecutive deliveries. Women with PTD in the first pregnancy were excluded. Characteristics and complications of the first pregnancy were compared among cases, defined as women with PTD in their second pregnancy, and the controls, defined as women delivering at term in their second pregnancy. A multivariable logistic regression model was used to study the association between pregnancy complications (in the first pregnancy) and PTD (in the subsequent pregnancy), while adjusting for maternal age and the interpregnancy interval. (3) Results: A total of 39,780 women were included in the study, 5.2% (*n* = 2088) had PTD in their second pregnancy. Women with PTD, as compared to controls (i.e., delivered at term in second pregnancy), were more likely to have the following complications in their first pregnancy: perinatal mortality (0.4% vs. 1.0%), small for gestational age (12.4% vs. 8.1%), and preeclampsia (7.6% vs. 5.7%). In the multivariable model, after adjusting for maternal age, interpregnancy interval and co-morbidities, having any one of these first pregnancy complications was independently associated with an increased risk for PTD (adjusted OR = 1.44; 95%CI 1.28–1.62), and the risk was greater if two or more complications were diagnosed (adjusted OR = 2.09; 95%CI 1.47–3.00). These complications were also risk factors for early PTD (<34 gestational weeks), PTD with a systematic infectious disease in the background, and possibly with spontaneous PTD. (4) Conclusions: First pregnancy complications are associated with an increased risk for PTD in the subsequent pregnancy. First pregnancy, although ending at term, may serve as a window of opportunity to identify women at risk for future PTD.

## 1. Introduction

Preterm delivery (PTD), defined as delivery before 37 complete gestational weeks [1], is the main cause for newborn death and childhood disability and the second cause of death in children up to the age of five years [2]. PTD rates vary by country, ranging in recent years from ~5% in European countries, 9.6% in the USA and 18% in some African countries [3,4]. In most countries, PTD rates are increasing, and an estimated 15 million babies (11.1% of live births), are born premature worldwide every year [2,3,5].

Fetal development occurs throughout the entire pregnancy until full term, therefore, when PTD occurs, the newborn is not physiologically and metabolically mature, leading to immediate and long term complications [3]. Risk and severity of these complications depend mainly on gestational age at delivery and increase with reduced gestational age.

The causes for PTD are mainly unknown, and they are usually multifactorial, including genetic factors, utero-placental dysfunction or underlying inflammation processes [6].

PTD risk factors, including subtypes of PTD, have been extensively studied. The leading PTD risk factor is having a history of PTD [7,8,9,10]. The risk increases with each additional PTD in a woman’s history, or if the PTD occurred in the immediately preceding pregnancy, and with earlier gestational age at the previous PTD [7].

It is recommended for women with a history of PTD to receive more intensive prenatal monitoring, including treatment strategies to reduce PTD risk. Often weekly mid-trimester 17-alpha-hydroxyprogesterone caproate (17-OHPC) are applied to reduce risk for PTD recurrence [11,12].

A history of pregnancy complications in term pregnancies has also been suggested to be associated with subsequent pregnancy PTD risk [13,14,15]. These complications mainly include threatened PTD, small for gestational age (SGA) and perinatal mortality. Less is known, however, regarding the risk factors by the type of PTD and by the extremity of the PTD.

Since a history of PTD is a main risk factor for its recurrence, the aim of the current study was to identify additional PTD risk factors, among a population of women without a history of PTD. Specifically, the risk factors were studied among women with spontaneous PTD, early PTD (<34 gestational weeks), and PTD with an infectious disease in the background (with a possible inflammatory etiology).

## 2. Experimental Section

The study was conducted at the Soroka University Medical Center (SUMC) located in the Southern region of Israel. SUMC, the single tertiary medical center in the region, serves a population of >1 million residents, and has the country’s largest birthing center with approximately 17,000 yearly births in recent years.

The study protocol received the SUMC IRB approval (#0355819SOR, October 2019), and informed consent was exempt.

A retrospective population-based nested case−control study was conducted. Inclusion criteria: All women with two first singleton consecutive deliveries between the years 1988–2017. Exclusion criteria: Women with PTD in the first pregnancy, multiple gestations (in either pregnancy). Cases were defined as women with PTD in their second pregnancy, and they were compared to the controls, defined as women delivering at term in their second pregnancy. Primary outcomes: Characteristics and complications of the first pregnancy, which were compared among cases and controls.

First pregnancy characteristics and complications that were significantly different between cases and controls were included in the multivariable analysis. Multivariable logistic regression models were used to study the association between pregnancy complications (in the first pregnancy) and PTD (in the subsequent pregnancy), while adjusting for maternal age (3 categories: <20; 20–35; ≥35) and interpregnancy interval (3 categories: <6 months; 6 months- 5 years; ≥5 years). The interpregnancy interval was defined as the time between first delivery and best estimation of first day of last menstruation period of the second pregnancy, based on clinical evaluation and first trimester sonar test. Interpregnancy interval, either short or long, has been associated with increased risk for several adverse pregnancy outcomes, including small for gestational age (SGA, defined as birthweight < 5th percentile for gestational age and sex), low birthweight (<2500 g), PTD and perinatal death [16,17,18,19,20]. It has also been shown to affect offspring long-term health [21,22]. The short interpregnancy interval may not allow the mother enough time to recuperate physiologically from the previous pregnancy and birth, and increase the risk for SGA and PTD. A long interpregnancy interval is associated with older maternal age, obesity and increased incidence of secondary infertility, morbidities and pregnancy complications.

A combined adverse pregnancy score was created, which summed the following first pregnancy complications (which were associated with second pregnancy preterm birth, based on the first step analysis): SGA, perinatal mortality or preeclampsia (defined as either of the following ICD-9 codes: 642.41; 642.42; 642.51; 642.52; 642.61; 642.62). Scoring of this variable ranged between 0 = no complications; 1 = one complication, 2 = two or more complications. A multivariable logistic model was then used to study whether the risk for PTD in the second pregnancy increased with each first pregnancy complication, and for each additional complication, based on the combined adverse pregnancy score. Women without first pregnancy complications were defined as the reference group.

In order to evaluate how early the second pregnancy PTD occurred following the first pregnancy complications, the mean (±standard deviation, SD) of gestational ages among the cases were evaluated regarding each first pregnancy complication.

Several subanalyses were performed that were:

1. Aiming to study risk factors for spontaneous PTD, the incidence of first pregnancy complications was compared between term deliveries and cases of spontaneous PTD in the second pregnancy. Spontaneous PTD included the following diagnoses: spontaneous premature rupture of the membranes or premature contractions (ICD-9 codes: 651.1; 644.21, respectively). Excluded were all cases of indicated PTD or PTD of a nondefinite nature (they may have been either spontaneous or indicated), which had the following diagnoses: placental abruption (ICD-9 code: 641.21), fetal growth restriction, preeclampsia (ICD-9 codes: 642.41, 642.51, 642.61, 642.62, 642.42, 642.52), meconium stained amniotic fluid (ICD-9 code 656.81), chronic hypertension (ICD-9 code 642.01), polyhydramnios (ICD-9 code 657.01), oligohydramnios (ICD-9 code 658.01), or pathological presentation (breech, transverse of face, ICD-9 codes 652.81, 652.21, 652.2, 652.31, 652.41, 660.31).

2. Aiming to study risk factors for early PTD (<34 weeks), a more severe outcome with greater complications to the offspring, incidence of first pregnancy complications was compared between term deliveries and cases of early PTD in second pregnancy. Women who delivered between 34.0–36.99 gestational week were excluded.

3. Aiming to study risk factors for PTD involving an infectious etiology, incidence of first pregnancy complications was compared between term deliveries and PTD cases involving an infection etiology in the second pregnancy. Systematic infection was defined as having either of the following, prior to the PTD: bacterial or viral infections, of known or unknown causes, including pneumonia, urinary tract infections, endometritis, etc.

The multivariable analysis with the combined adverse score was performed for each of the subanalyses.

## 3. Results

A total of 39,780 women were included in the study, 5.2% (*n* = 2088) delivered preterm in their second pregnancy (i.e., cases). Of them, the incidence of definite spontaneous PTD (following premature rupture of the membranes or contractions) was 14.8% (*n* = 310) and incidence of indicated PTB (following premature rupture of the membranes, fetal growth restriction or preeclampsia) was 15.2% (n = 318).

Table 1 presents a comparison of the participants' characteristics, as well as first and second pregnancy characteristics, between cases and controls. As can be seen, cases were slightly older, delivered heavier newborns, and were more likely to have the following complications in their first pregnancy: perinatal mortality (1.0% vs. 0.4%; OR = 2.56 95%CI 1.60–4.09, *p* < 0.001), small for gestational age (SGA, 12.4% vs. 8.1%; OR = 1.60 95%CI 1.39–1.83, *p* < 0.001), preeclampsia (7.6% vs. 5.7%; OR = 1.34 95%CI 1.14–1.59, *p* < 0.001) and offspring with low birthweight (LBW, birthweight <2500 gr.: 12.3% vs. 5.7%; OR = 2.31; 2.01–2.65, *p* < 0.001). Rates of gestational diabetes, placental abruption and cesarean deliveries were comparable between the groups. Among the cases of first pregnancy perinatal mortality, the possible causes of the mortality did not differ between the cases and the controls, for instance: 7 (35%) versus 44 (31%) were diagnosed with chromosomal abnormalities or malformations among cases and controls, respectively, and intrauterine fetal death 11 (55.0%) versus 72 (50.7%) among cases and controls, respectively.

In the second pregnancy, the incidence of preeclampsia, fetal growth restriction and placental abruption were all higher among the pregnancies ending with PTD as compared to term deliveries.

In three multivariable models (not presented), which adjusted for categories of maternal age in second pregnancy and interpregnancy interval, first pregnancy with either SGA (adjusted OR = 1.54; 95%CI 1.35–1.77, *p* < 0.001), preeclampsia (adjusted OR = 1.36; 95%CI 1.15–1.61, *p* = 0.001) or perinatal mortality (adjusted OR = 2.27; 95%CI 1.41–3.64, *p* < 0.001), was independently associated with second pregnancy PTD risk. A combined adverse first pregnancy outcome variable was created, including the sum of the following diagnoses: SGA, perinatal mortality and preeclampsia (scoring 0, 1, ≥2). In the multivariable model presented in Table 2, having a history of any one of the complications was independently associated with an increased risk for PTD (adjusted OR = 1.46; 95%CI 1.30–1.65, *p* < 0.001), and the risk was greater if two or more complications were diagnosed (adjusted OR = 2.20; 95%CI 1.54–3.13, *p* < 0.001). The model was adjusted for maternal age, interpregnancy interval, maternal comorbidities and year of delivery.

The distribution of gestational ages among PTD in second pregnancy, by first pregnancy complication were as follow: among pregnancies with SGA 33.81 ± 3.1; among pregnancies with preeclampsia 34.2 ± 3.1; and among PTD following pregnancies with perinatal mortality 33.2 ± 3.7.

There were 310 (14.8%) definite spontaneous PTD and 318 (15.2%) cases of indicated PTD. In the subanalysis, cases of spontaneous PTD were compared to the control group (term delivery). The incidence of first pregnancy SGA, perinatal mortality and preeclampsia were all higher among the spontaneous PTD group, the differences were not statistically significant, most likely due to the small sample size (SGA: 11.9% versus 9.0%, OR = 1.36, 95%CI 0.98–1.99, *p* = 0.071, perinatal mortality: 0.6% versus 0.4%, OR = 1.72; 95%CI 0.42–6.96, *p* = 0.33, preeclampsia: 6.1% versus 5.7%, OR = 1.07; 95%CI 0.67–1.71, *p* = 0.73). A combined adverse first pregnancy outcome variable was created, including any of the following diagnoses: SGA, perinatal mortality or preeclampsia (scoring 0 or 1). The incidence of having at least one complication in the first pregnancy was 19.0% versus 15.4% among the spontaneous PTD versus the term pregnancies, respectively, (OR = 1.29, 95%CI 0.97–1.72, *p* = 0.08). In the multivariable analysis (presented in Table 3), while adjusting for maternal age and interpregnancy intervals, first term delivery with either SGA, perinatal mortality or preeclampsia, was associated with an increased risk for spontaneous subsequent pregnancy PTD (adjusted OR = 1.29, 95%CI 0.97–1.72, *p* = 0.078). This finding was not statistically significant, however the possibility that the findings were due to insufficient power cannot be ruled out (Power = 39%).

There were 543 (1.4%) cases of early preterm (gestational age < 34) in second delivery, compared to term deliveries. First pregnancy incidence of SGA (13.6% versus 8.3%, OR = 1.75, 95%CI 1.37–2.24, *p* < 0.001) and perinatal mortality (1.1% versus 0.4%, OR = 2.80, 95%CI 1.23–6.35, *p* = 0.024) were both significantly higher among women with second pregnancy early preterm delivery. The incidence of preeclampsia was slightly higher (6.6% versus 5.8%, OR = 1.5, 95%CI 0.82–1.61) among this group. Among mothers without a history of any complications, the risk for early PTD was 1.3%, and the risk was 2.3% and 3.2% among mothers with one and two complications, respectively (*p* for trend < 0.001). In the multivariable analysis (presented in Table 3), while adjusting for maternal age and interpregnancy intervals, first term delivery with either SGA, perinatal mortality or preeclampsia, was associated with an increased risk for early subsequent pregnancy PTD (adjusted OR = 1.73; 1.42–2.11, *p* < 0.001).

There were 215 (8.8% of all PTDs) PTD second deliveries with systematic infectious disease in the background, compared to 37,692 term deliveries. Among this group 40 (18.6%) had definite spontaneous delivery. First pregnancy incidence of SGA (11.6% versus 8.1%, OR = 1.49, 95%CI 0.98–2.26, *p* = 0.073), perinatal mortality (1.9% versus 0.4%, OR = 5.01, 95%CI 1.84–13.67, *p* = 0.01) and preeclampsia (9.8% versus 5.7%, OR = 1.78, 95%CI 1.13–2.79, *p* = 0.018) were all higher among the cases with second pregnancy PTD with systematic infectious disease in the background. Among mothers without a history of any complications, the risk for PTD with systematic infectious disease in the background was 0.5%, and the risk was 0.8% and 2.0%, among mothers with any one or ≥2 complications, respectively (*p* for trend < 0.001). In the multivariable analysis (presented in Table 3), while adjusting for maternal age and interpregnancy intervals, first term delivery with either SGA, perinatal mortality or preeclampsia, was associated with an increased risk for PTD in second pregnancy with systematic infectious disease in the background (adjusted OR= 1.63; 1.17–2.28, *p* = 0.004).

## 4. Discussion

In this large population-based retrospective nested case−control study, first term pregnancy complicated with either SGA, preeclampsia or perinatal mortality, was associated with an increased risk for PTD in the subsequent pregnancy. These findings were also true specifically for early PTD, PTD with a possible inflammatory etiology, or spontaneous PTD, although the later was without statistical significance. This association was independent of maternal age, interpregnancy interval and maternal comorbidities. Exposure to more than one of these first pregnancy complications was associated with an even greater risk.

First pregnancy complications were associated with not only late, near term PTD, but also with extreme PTD: while the majority of second pregnancy PTD occurred between gestational ages 35–36, among women with a first pregnancy which ended with perinatal mortality, 25% of PTD deliveries occurred at <32 gestational weeks; and in nearly 40% of women with first pregnancy SGA, the PTD occurred at <34 gestational weeks. The associations between the first pregnancy complications were weaker regarding spontaneous PTD, which suggest other risk factors are relevant in these cases, however the possibility of lack of power to detect such differences cannot be ruled out.

Findings of the current study are in agreement with previous studies that found that women with a history of term SGA or fetal mortality were at increased risk for PTD [13]. The strongest risk factor of PTD is a previous PTD, therefore chronic environmental [23,24,25], and genetic factors are most likely involved in PTD etiology [26,27]. The current study addresses first PTD occurrence and its association with previous term pregnancy SGA, preeclampsia and perinatal mortality, all of which may be due to several mechanisms and causes. These four complications may share similar mechanisms, and therefore reoccur in the same mother, and can serve as markers of increased risk for the other complications [28].

The underlining cause and mechanism of PTD is not yet completely understood, and even less is known regarding causes of spontaneous PTD. The main mechanisms that have been suggested are inflammation, infection, and vascular pathologies [29,30]. Usually multiple etiologies are involved, including: cervical insufficiency, decline in progesterone action and insufficiency or ischemic placental−uterine unit [29,31,32]. Impaired placental implantation processes and insufficient fetal nutrition and growth may cause deliveries of SGA newborns, preeclampsia, perinatal mortality, placental abruption and PTD [33,34]. In the current study, placental abruption in the second pregnancy was a risk factor for PTD in the second pregnancy, but having a history of term placental abruption was not a risk factor.

An inflammatory process has been suggested as a main factor in PTD, causing premature contractions and (mainly) spontaneous PTD [35]. In our study, women delivering preterm with an infectious disease in the background, have also presented with higher rates of SGA, preeclampsia and perinatal mortality in the first pregnancy. Although it is not clear whether neonatal outcomes are affected by the maternal infection [36], it is possible women with an infectious disease during pregnancy, and with a history of these complications, would benefit from PTD prevention strategies.

Several study limitations need to be addressed. Since this was a retrospective cohort and based on medical records, data regarding additional potential confounding variables was unavailable, such as environmental and life-style characteristics, and may have caused a residual confounding effect. However, since this was a large population-based study, in which the two pregnancies of each mother were matched and compared, it can be expected that familial, background and environmental factors were relatively similar between the two pregnancies, and in case a distortion of the true association existed, it was minimal.

The aim in the current study was to identify second pregnancy PTD risk factors during the first pregnancy, and therefore the current findings are not valid or relevant for PTD risk among primiparous women or for PTD recurrence. Still, according to our findings, initial PTD occurred in 5.2% of second pregnancies, therefore this is a relatively prevalent pregnancy complication, to which our findings are relevant.

It is possible women with previous pregnancy complications were under more frequent and closer monitoring and were therefore more likely to be diagnosed with second pregnancy complications, leading to a higher incidence of indicated PTD among this group. However, a subanalysis among spontaneous second pregnancy PTD showed similar results, suggesting detection bias is unlikely.

PTD is a major cause of death and a significant cause of long-term morbidities and disabilities, and the risks are greater with decreasing gestational age at delivery. Lowering the rate of this major pregnancy complication has been declared by the World Health Organization as “an urgent priority for reaching the Millennium Development Goal, calling for the reduction of child deaths” [2]. While risk factors for PTD have been widely studied, and although strategies and diagnostic tools to prevent PTD have been practised for over 30 years, the expectations have not been met and PTD rates have not declined. Some PTD risk factors are preventable, and addressing them, in the personal and population levels, may decrease PTD risk. Even a small reduction in PTD can have a large public health and economic impact, both in terms of preventing perinatal mortality, morbidity and lifelong disability among affected infants.

## 5. Conclusions

First pregnancy complications are associated with an increased risk for PTD in the subsequent pregnancy, and specifically with PTD with maternal infectious disease in the background, early PTDs, and possible spontaneous PTDs. First pregnancy, although ending at term, may serve as a window of opportunity to identify women at risk for future PTD, and PTD prevention strategies which are recommended for women with a history of PTD, should be considered for women with history of these other pregnancy complications.

## Figures and Tables

**Table 1:** Maternal, first and second pregnancy characteristics by cases and controls.

	Cases (PTD Second Pregnancy) *n* = 2088 (5.2%)	Controls (Term Second Pregnancy) *n* = 37,692 (94.8%)	*p*-Value
Maternal Characteristics			
Ethnicity			<0.001
Jewish	928 (4.4)	20,036 (95.6)	
Bedouin	1160 (6.2)	17,656 (93.8)	
Smoking	23 (1.1)	458 (1.2)	0.75
Chronic hypertension	32 (1.5)	217 (0.6)	<0.001
Diabetes mellitus	13 (0.6)	90 (0.2)	0.003
First Pregnancy Characteristics			
Maternal age (mean ± SD)	23.39 ± 4.0	22.72 ± 4.2	<0.001
<20	393 (18.9)	4564 (12.1)	<0.001
20–35	1624 (78.0)	32,231 (85.6)	
≥35	65 (3.1)	843 (2.2)	
Birthweight (mean ± SD)	3139 ± 416	2961 ± 425	<0.001
Gestational age (mean ± SD)	38.93 ± 1.30	39.49 ± 1.25	<0.001
First and second pregnancy interval mean ± SD)	1.56 ± 1.52	1.47 ± 1.75	0.03
<6 months	582 (27.9)	7550 (20.0)	<0.001
between 6 months and 5 years	1414 (67.7)	28,810 (76.4)	
≥5 years	92 (4.4)	1332 (3.5)	
			Odds ratio; 95%CI, *p*-value
Fertility treatments *	60 (2.9)	1043 (2.8)	1.04; 0.8–1.35, 0.74
Obesity *	10 (0.5)	275 (0.7)	0.66; 0.35–1.23, 0.23
Cesarean delivery	241 (11.5)	4494 (11.9)	0.96; 0.84–1.10, 0.62
full dilatation cesarean sections	44 (18.3)	596 (13.3)	1.46; 1.04–2.05, 0.03
Low Apgar (<7) at 5 min	9 (0.4)	179 (0.5)	0.91; 0.47–1.79, 1.0
Perinatal mortality	20 (1.0)	142 (0.4)	2.56; 1.60–4.09, <0.001
Low birthweight (<2500 gr.)	256 (12.3)	2148 (5.7)	2.31; 2.01–2.65, <0.001
Chromosomal abnormalities or congenital malformations	130 (6.2)	2065 (5.5)	1.14; 0.95–1.37, =0.15
Small for gestational age *	258 (12.4)	3059 (8.1)	1.60; 1.39–1.83, <0.001
Mild or severe preeclampsia or eclampsia	158 (7.6)	2165 (5.7)	1.34; 1.14–1.59, <0.001
Gestational diabetes	67 (3.2)	1096 (2.9)	1.11; 0.86–1.42, 0.43
Placental abruption	9 (0.4)	100 (0.3)	1.63; 0.82–3.22, 0.19
Prolonged first stage of delivery	38 (1.8)	1031 (2.7)	0.66; 0.47–0.91, 0.012
Prolonged second stage of delivery	81 (3.9)	1401 (3.7)	1.04; 0.83–1.31, 0.68
Second Pregnancy Characteristics			
Preeclampsia	110 (5.3)	764 (2.0)	2.69; 2.19–3.30, <0.001
Fetal growth restriction	161 (7.7)	504 (1.3)	6.16; 5.13–7.40, <0.001
Placenta abruption	75 (3.6)	84 (0.2)	16.68; 12.18–22.85, <0.001
Rupture of the membranes	300 (14.4)	2518 (6.7)	2.34; 2.06–2.67, <0.001

* Fertility treatments: including ovulation induction or in vitro fertilization; obesity: BMI > 30; small for gestational age: birthweight < 5th percentile for gestational age and gender.

**Table 2:** Multivariable analysis for the association between first pregnancy complications and PTD risk in second pregnancy.

Variable	Adjusted Odds Ratio; 95%CI	*p*
Any adverse first pregnancy outcome (vs. none) *	1.44; 1.28–1.62	<0.001
Any two or three complications (vs. none)	2.09; 1.47–3.00	<0.001
Maternal age		
<20	1.56; 1.38–1.75	<0.001
20–35	1 (Ref.)	
≥35	1.16; 0.91–1.48	0.216
Interpregnancy interval		
<6 months	1.44; 1.30–1.60	<0.001
between 6 months and 5 years	1 (Ref.)	
≥5 years	1.44; 1.15–1.80	0.001
delivery year	1.0; 0.99–1.00	0.459
obesity	0.81; 0.29–2.27	0.694
chronic high blood pressure	2.53; 1.73–3.70	<0.001
diabetes	2.65; 1.46–4.79	0.001

* The following complications were included: SGA, perinatal mortality or preeclampsia.

**Table 3:** Multivariable analysis for the association between first pregnancy complications and PTD risk in second pregnancy.

	Spontaneous PTD Only		Early PTD Only		PTD Following a Systemic Infectious Disease	
Variable	Adjusted Odds Ratio; 95%CI	*p*	Adjusted Odds Ratio; 95%CI	*p*	Adjusted Odds Ratio; 95%CI	*p*
Any adverse first pregnancy outcome (vs. none) *	1.29; 0.97–1.72	0.078	1.73; 1.42–2.11	<0.001	1.63; 1.17–2.28	0.004
Maternal age						
<20	0.99; 0.7–1.42	0.98	2.06; 1.67–2.54	<0.001	1.51; 1.05–2.17	0.027
20–35	1 (Ref.)		1 (Ref.)		1 (Ref.)	
≥35	1.41; 0.83–2.38	0.20	1.51; 0.99–2.32	0.06	0.97; 0.45–2.11	0.95
Interpregnancy interval (years)						
<6 months	0.95; 0.71–1.28	0.75	1.39; 1.15–1.70	0.002	1.13; 0.82–1.57	0.45
between 6 months and 5 years	1 (Ref.)		1 (Ref.)		1 (Ref.)	
≥5 years	1.64; 1.01–2.66	0.047	1.36; 0.88–2.12	0.16	1.46; 0.76–2.83	0.26

* The following complications were included: SGA, perinatal mortality or preeclampsia.

## Data Availability

Data will be available by request and according to the IRB restrictions.
